# Comparable long‐term functional outcomes of subvastus and medial parapatellar approach in total knee arthroplasty: A 10‐year follow‐up study

**DOI:** 10.1002/jeo2.70035

**Published:** 2024-10-08

**Authors:** Pooya Hosseini‐Monfared, Alireza Mirahmadi, Shayan Amiri, Reza Minaie, Mohammad Hossein Ghafouri, Seyed Morteza Kazemi

**Affiliations:** ^1^ Bone Joint and Related Tissues Research Center Shahid Beheshti University of Medical Sciences Tehran Iran; ^2^ Shohadaye Haftom‐e‐Tir Hospital Iran University of Medical Sciences Tehran Iran

**Keywords:** KSS, medial parapatellar, subvastus, total knee arthroplasty, WOMAC

## Abstract

**Background:**

Surgeons usually use the medial parapatellar or subvastus approaches for total knee arthroplasty (TKA). The subvastus approach is rapidly gaining recognition to reduce damage to the extensional mechanism and recover faster after surgery. This study compares the long‐term outcomes of the conventional medial parapatellar and subvastus approaches in primary TKA during a minimum 10‐year follow‐up.

**Methods:**

In a retrospective longitudinal follow‐up study from 2008 to 2013, 60 eligible patients for primary TKA were included. The patients were divided into two groups: one undergoing TKA with the subvastus approach (*n* = 30) and the other with the conventional medial parapatellar approach (*n* = 30). Postoperatively, the patients were followed up for 10–15 years. Patients were assessed using the Western Ontario and McMaster Universities Osteoarthritis Index (WOMAC), Knee Society Score (KSS), and Visual Analogue Scale index for pain.

**Results:**

The time required to perform an active straight leg raise (SLR) was significantly shorter in the subvastus group (*p* < 0.001) at early postoperation evaluation. Patients in the subvastus group had lower pain and better knee functional scores at the one‐year follow‐up (*p* < 0.05). There was no difference between the two groups regarding duration of hospitalisation, blood loss, operation time, length of the scar, and postoperative complications. Both approaches had similar long‐term results regarding pain and functional scores of WOMAC (6.2 ± 1.2 vs. 6.3 ± 1.3, *p*‐value = 0.69) and KSS scores (93.1 ± 6.8 vs. 95.0 ± 3.2, *p*‐value = 0.42).

**Conclusion:**

The subvastus approach was associated with a shorter time to achieve active SLR, higher functional scores, and better pain relief at early postoperative evaluations. However, both techniques had similar long‐term outcomes in terms of pain and functional scores, as measured by the WOMAC and KSS scales.

**Level of Evidence:**

II

**LIST OF ABBREVIATIONS**
BMI: body mass index
KA: kinematic alignment
KFS: Knee Function Score
KSS: Knee Society Score
LMWH: low molecular weight heparin
MA: mechanical alignment
MCL: medial collateral ligament
MP: medial parapatellar
OA: osteoarthritis
OKS: Oxford Knee Score
PJI: periprosthetic joint infection
ROM: range of motion
SLR: straight leg raise
SSI: surgical site infections
SV: subvastus
TKA: total knee arthroplasty
VAS: Visual Analogue Scale
WOMAC: Western Ontario and McMaster Universities Osteoarthritis Index

## INTRODUCTION

Total knee arthroplasty (TKA) is considered an effective treatment for end‐stage osteoarthritis (OA), providing pain relief, restoring joint function, and enhancing overall quality of life [1]. Different approaches have been proposed for TKA. The surgical approach for TKA affects the degree of soft tissue disruption, quadriceps muscle trauma, patellar eversion or subluxation, and ultimate implant positioning and alignment [2, 3].

Over the years, the surgical approaches employed in TKA have improved, resulting in high satisfaction among most patients who receive this form of treatment [4]. The most commonly used technique is the medial parapatellar approach, first described by Von Langenbeck in 1879, providing a good surgical field visualisation for installing prosthetic components [5, 6]. However, this approach could damage the quadriceps tendon and possibly form painful scar tissue, leading to a weakening of the extensor mechanism. Moreover, an incision near the patella may damage the vascularity of the patella, which induces anterior knee pain and patellar fracture [7, 8, 9].

In 1929, the subvastus approach was introduced in German literature, which was later brought into English literature by Hofmann in 1991 as a less invasive approach [10, 11]. Evidence has shown that minimal‐invasive approaches in TKA are effective alternatives to the medial parapatellar approach [12, 13]. The subvastus approach preserves the intact patella's vascular supply and maintains the knee's extensor mechanism. The subvastus approach was shown to be associated with minimised soft tissue damage and pain, faster recovery, early movement of joints, and increased joint function [14, 15, 16].

Some studies have recommended the subvastus approach because of satisfactory outcomes such as straight leg raising (SLR) during the early postoperative period, reduced blood loss and opioid consumption, and facilitation of knee joint flexion during the recovery period [17, 18, 19]. However, other studies have reported some complications for the subvastus approach, such as component malalignment, skin injuries, haematoma, and peroneal nerve injury, leading to concerns among surgeons [2, 20, 21, 22, 23, 24, 25]. Nevertheless, based on the literature, there has been no research on the long‐term effects of these two methods. This study compares the conventional medial parapatellar and subvastus approaches in primary TKA after a minimum of 10 years of follow‐up to clarify which procedure has better long‐term results. We hypothesised that the long‐term functional outcomes would be similar between the two approaches.

## MATERIAL AND METHODS

### Study Characteristics

In a longitudinal follow‐up study between September 2008 and December 2013, we evaluated 143 TKAs performed by a single surgeon at our institution. Inclusion criteria consisted of patients who were diagnosed with late‐stage knee OA and were candidates for primary unilateral TKA. Exclusion criteria included: history of previous knee surgery, contracture of lower limbs, neuromuscular disease, acute spinal disorders, contralateral knee replacement, knee joint infection, knee varus or valgus more than 10 degrees, knee flexion angle exceeding 15 degrees, post‐traumatic arthrosis, and history of rheumatoid arthritis [26]. Of the 143 TKAs performed by the single surgeon, 12 were revision surgeries and were consequently excluded. Only 78 of these TKAs were performed with the same prosthesis (Scorpio NRG, Stryker). Furthermore, ten patients underwent TKA due to etiologies other than osteoarthritis, and we were unable to contact eight of the patients for long‐term follow‐up. Finally, 60 patients were included in the study. Approval was acquired from the institutional ethics committee (IR.SBMU.MSP.REC.1397.694), and patients were asked to fill out a written informed consent form at the last follow‐up.

### Surgical Techniques

Of the 60 patients included in the study, 30 were assigned to the subvastus (SV) and 30 to the medial parapatellar (MP) groups. In the MP approach, the skin was incised with a longitudinal midline incision from the proximal to the superior pole of the patella and extending to the tibial tuberosity. The incision was continued between the vastus medialis and rectus femoris muscles, and a medial parapatellar arthrotomy was performed by incising the medial patellar retinaculum and opening the joint capsule.

The subvastus approach was performed according to the method described by Hofmann et al. [11]. In the SV approach, a straight anterior midline skin incision was made from the proximal to the superior pole of the patella and extended to the tibial tuberosity, similar to the MP group. The inferior edge of the vastus muscle was found and separated from the periosteum and intermuscular septum using gentle dissection, approximately 10 cm proximal to the adductor tubercle. The connection to the patella was exposed by pulling the muscle forward, and an arthrotomy was performed by incising the synovium proximally and the capsule distally.

All procedures were performed under standard spinal anaesthesia with a tourniquet from the beginning of the skin incision to the end of the retinacular closure. Intraoperative sedation was provided based on the anesthesiologist's discretion using a 7–10 mL/h infusion of propofol 2% (Fresenius Kabi Austria Gmbh).

A skilled surgeon performed all surgeries using the same cemented posterior stabilised prosthesis (Scorpio NRG, Stryker) for all patients. Patellar resurfacing was not performed in any of the cases. Drains were removed after 24 h. Patients received intravenous antibiotics (Cefazolin) for 3 days after the surgery and then oral antibiotics for 5 days. Prophylactic low molecular weight heparin (LMWH) was used for 10–14 days to prevent thromboembolism. Patients were allowed to take acetaminophen (maximum 2 g per day) if they had a pain Visual Analogue Scale (VAS) > 3. A standardised physical therapy protocol began on postoperative day one, and patients were encouraged to walk as much as possible with supervision from nursing staff or their families. Patients were discharged after full rehabilitation.

### Outcome Measures

This study's primary outcome measure was the patients' long‐term functional outcomes. Knee functions were assessed by the Knee Society Score (KSS) and Western Ontario and McMaster Universities Osteoarthritis (WOMAC) index before surgery, one year after surgery, and at the final follow‐up, 10–15 years after surgery [27, 28]. Also, the VAS pain scores were recorded at the same follow‐up periods.

The knee range of motion (ROM) and the SLR were chosen as the evaluation indices for the extensor mechanism rehabilitation. Time to active SLR was evaluated during the postoperative hospitalisation period, and knee ROM was assessed 12 months after the surgery. We recorded the patients' basic demographic characteristics and evaluated the operation time, tourniquet time, length of the skin incision, blood transfusion, total bleeding volume, and length of the hospital stay.

Potential surgical complications, including superficial surgical site infections (SSI), bleeding, neurovascular complications, haematoma formation, thromboembolic events, wound complications, periprosthetic joint infection (PJI), patellar fractures, and periprosthetic fractures were recorded.

### Statistical Analysis

Statistical analysis was performed using SPSS statistical software version 29 and GraphPad Prism version 8 (GraphPad Prism Software Inc.). The Kolmogorov–Smirnov test was used to check the normality of the variables. Students' *t*‐tests were used to analyse normally distributed continuous variables. In addition, we used the Mann–Whitney *U* test for analysing not normally distributed continuous variables. The Pearson Chi‐square test was used for categorical variables analysis. *P* values less than 0.05 were considered statistically significant. Power analysis utilizing the WOMAC scores reported by Aladrii et al. in 2024 indicated that our study would achieve a statistical power of 87% [29].

## RESULTS

In total, 60 patients were evaluated in this study and assigned into two groups, including 30 in the MP group and 30 in the SV group. The mean follow‐up time was 11.6 ± 1.5, and the range of follow‐up time was from 10 to 15 years. There were 26 females (86.6%) in the MP group and 24 females (80%) in the SV group (*p* = 0.48). There was also no significant difference between groups regarding age, gender, and BMI (*p* > 0.05). Both groups had similar preoperative pain and functional scores (*p* > 0.05) (Table 1).

**Table 1:** Preoperative pain and functional scores.

Variable	MP group	SV group	*p*‐Value
	(*n* = 30)	(*n* = 30)	
Knee ROM (degree)	84.7 ± 8.1	84.0 ± 7.3	0.94
VAS	8.6 ± 1.0	8.8 ± 0.6	0.26
WOMAC			
Pain	15.4 ± 3.2	14.4 ± 2.8	0.46
Stiffness	6.5 ± 2.1	6.7 ± 1.4	0.73
Physical function	36.3 ± 11.7	38.6 ± 10.4	0.28
Overall score	58.2 ± 11.5	59.7 ± 8.6	0.51
KSS	50.3 ± 7.2	53.1 ± 8.1	0.24
KFS	53.4 ± 4.3	54.4 ± 6.5	0.35

Abbreviations: KFS, Knee Function Score; KSS, Knee Society Score; MP, medial parapatellar; ROM, range of motion; SV, subvastus; VAS, Visual Analogue Scale; WOMAC, Western Ontario and McMaster Universities Osteoarthritis.

Operation time and length of hospitalisation were not significantly different between the two groups (Table 2). The subvastus group had higher total blood loss than the MP group, but the difference was not statistically significant (602 ± 85.9 vs. 586 ± 98.4, *p*‐value = 0.61). Patients in the SV group performed the active straight leg raise (SLR) test significantly faster than the MP group (*p* < 0.001).

**Table 2:** Operative and early postoperative variables between the two groups (mean ± SD).

Variable	MP group	SV group	*p*‐Value
	(*n* = 30)	(*n* = 30)	
Operation time (min)	78.7 ± 4.6	74.3 ± 6.3	0.45
Tourniquet time (min)	54 ± 6.8	50 ± 4.7	0.46
Length of hospitalisation (day)	8.4 ± 0.3	8.3 ± 0.4	0.27
Time to active SLR (days)	3.6 ± 1.5	1.8 ± 1.3	<0.001*
Length of surgical scar (cm)	11.8 ± 0.7	12.1 ± 0.8	0.97
Total blood loss (mL)	586 ± 98.4	602 ± 85.9	0.61
Blood transfusion (mL)	367 ± 89	386 ± 74	0.37

Abbreviations: MP, medial parapatellar group; SLR, straight leg raise; SV, subvastus group.

* Statistically significant

**Figure 1:**
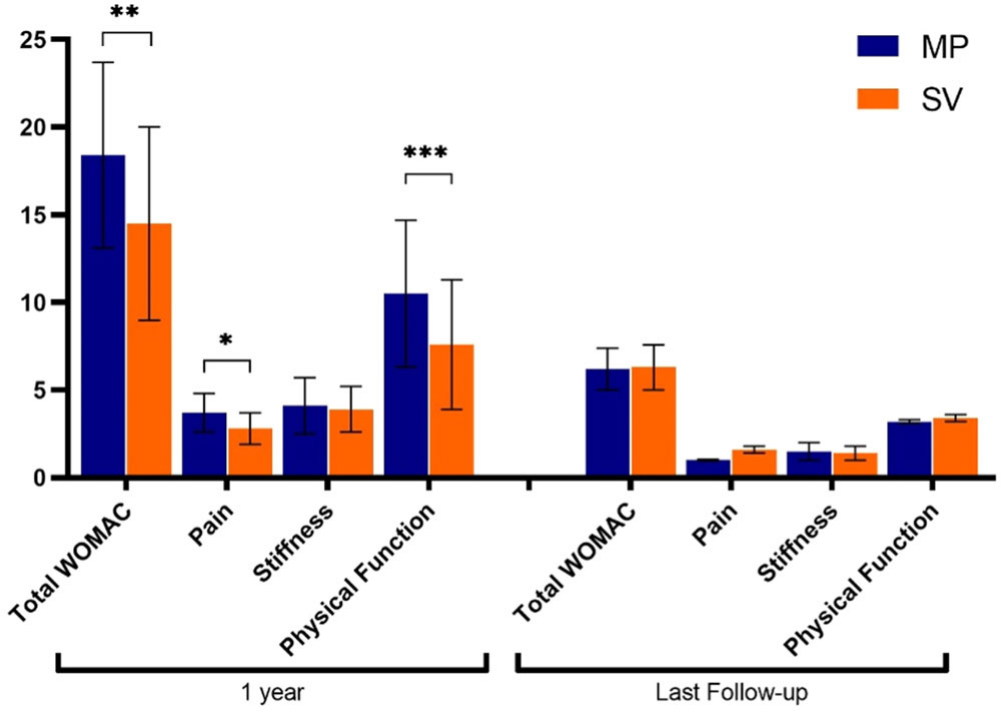
WOMAC scores during the follow‐up period in two groups. *: *p*‐value < 0.05, **: *p*‐value < 0.01, and ***: *p*‐value < 0.001. WOMAC, Western Ontario and McMaster Universities Osteoarthritis.

Knee ROM after 12 months of surgery was not significantly different between the two groups (MP group: 112.1 ± 18.6° vs. SV group: 111.1 ± 16.7°, *p*‐value = 0.86). The VAS pain scores were not significantly different between the two groups in the 12‐month and the last follow‐ups. Table 3 compares WOMAC subscales and overall scores between the two groups. They were significantly different in terms of pain, physical function, and overall scores one year after the operation (Figure 1). These scores were significantly lower in the subvastus group (*p* < 0.05). However, the results of the final follow‐up showed no significant difference regarding WOMAC subscales and overall scores (Figure 1).

**Table 3:** Postoperative pain and functional scores of the patients (mean ± SD).

Variables	MP group	SV group	*p*‐Value
	(*n* = 30)	(*n* = 30)	
1 year			
VAS pain	3.2 ± 0.6	2.9 ± 0.5	0.16
WOMAC			
Pain	3.7 ± 1.1	2.8 ± 0.9	**0.02** *
Stiffness	4.1 ± 1.6	3.9 ± 1.3	0.24
Physical function	10.5 ± 4.2	7.6 ± 3.7	**<0.001** **
Overall score	18.4 ± 5.3	14.5 ± 5.5	**0.008** **
KSS	83.0 ± 5.4	85.4 ± 3.8	0.09
KFS	83.7 ± 2.3	93.8 ± 7.1	**<0.001** **
Last follow‐up			
VAS pain	2.5 ± 0.4	2.5 ± 0.3	0.33
WOMAC			
Pain	1.0 ± 0.04	1.6 ± 0.2	0.25
Stiffness	1.5 ± 0.5	1.4 ± 0.4	0.40
Physical function	3.2 ± 0.1	3.4 ± 0.2	0.84
Overall score	6.2 ± 1.2	6.3 ± 1.3	0.69
KSS	93.1 ± 6.8	95.0 ± 3.2	0.42
KFS	94.5 ± 5.5	95.3 ± 6.1	0.44

Abbreviations: KFS, Knee Function Score; KSS, Knee Society Score; MP, medial parapatellar; SV, subvastus; VAS, Visual Analogue Scale, WOMAC, Western Ontario and McMaster Universities Osteoarthritis.

* *p* < 0.05

** *p* < 0.01.

**Figure 2:**
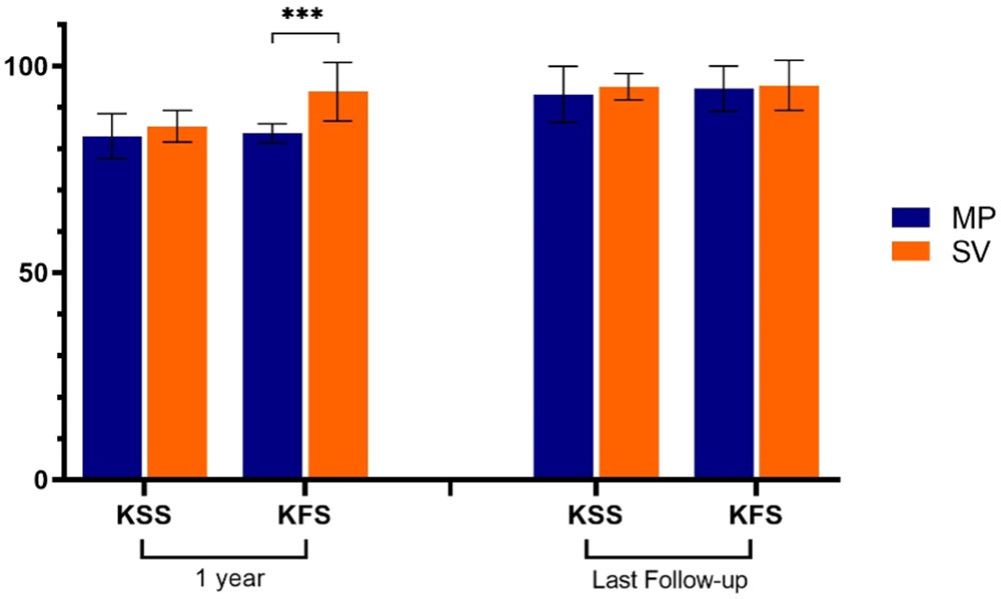
KSS and KFS scores during the follow‐up period in two groups. *: *p*‐value < 0.05, **: *p*‐value < 0.01, and ***: *p*‐value < 0.001. KFS, Knee Function Score; KSS, Knee Society Score.

The comparison of KSS scores between the two groups showed no significant difference at any time except for the functional score one year after the surgery, which was significantly better in the SV group (*p* < 0.001) (Figure 2, Table 3).

The SV and MP group had one and two superficial SSI, respectively. One patient in the SV group developed a myocardial infarction 12 weeks postoperatively. Venous thromboembolic events, pulmonary embolism (PE), neurovascular complications, wound complications, PJI, and periprosthetic fractures were not detected in any group.

## DISCUSSION

The subvastus approach was initially described in the first half of the 20th century, gaining surgeons' attention [11]. In the present study, we evaluated the short‐, mid‐, and long‐term functional outcomes between the subvastus and medial parapatellar groups. Our findings demonstrated that although the subvastus approach provides better early postoperative and midterm outcomes, the pain and functional scores of the patients were similar at the final follow‐up, 10–15 years after the surgery in the two groups. Patients in the subvastus approach had faster recovery of physical function, SLR, and less pain compared to the medial parapatellar approach. However, the evaluated outcomes and complications did not differ significantly between both methods at the final long‐term follow‐up. Several studies have reported subvastus short‐term postoperative outcomes and indicated significant advantages like decreased postoperative pain, preservation of patellar vascularity, improved patellar stability, diminished need for lateral release, better postoperative quadriceps control and strength, facilitation of rehabilitation, and shorter hospital stays in comparison with the medial parapatellar approach.

Surgeons are faced with numerous choices during TKA, each with its distinct advantages and disadvantages. Various techniques, such as kinematic and mechanical alignment (MA) techniques, have been proposed for TKA, and their early and late outcomes have been extensively evaluated in the literature [30, 31]. While initial studies suggested superior short‐term outcomes with kinematic alignment (KA) compared to MA, recent research has indicated that there are no significant differences between the two methods in long‐term follow‐up assessments after several years [32, 33, 34]. Additionally, surgeons encounter other conditions during surgery, such as managing iatrogenic medial collateral ligament injuries, with studies examining both short‐term and long‐term outcomes demonstrating no significant differences between treatment approaches [35]. Similar to the mentioned choices, surgeons performing the subvastus and mid‐parapatellar approaches require comprehensive assessments of both short‐term and long‐term results to guide decision‐making. While the literature has extensively compared short‐term and midterm (one to two years) follow‐up outcomes, there is a paucity of published data on long‐term follow‐up assessments. The subsequent section of this article will discuss the main findings from studies comparing short‐ and midterm follow‐up outcomes in TKA.

A study by Khan et al. comparing the subvastus and medial parapatellar approaches demonstrated that quadriceps muscle function assessed by time to active SLR was shorter in the subvastus group [36]. A systematic review and meta‐analysis study evaluating ten studies that compared the subvastus and medial parapatellar approaches demonstrated that the patients in the subvastus group had shorter days to active SLR compared to the medial parapatellar group with a mean difference of 1.9 [37]. Our findings aligned with the mentioned studies, and we observed a shorter time‐to‐SLR in the SV group. Furthermore, the meta‐analysis of the studies comparing the length of hospital stay and surgery duration between the two groups showed that these outcomes had statistically non‐significant differences [37]. We also found no significant difference between the two approaches concerning the surgery duration and length of hospital stay. A network meta‐analysis comparing the outcomes of different surgical approaches in TKA demonstrated that the subvastus approach was associated with higher postoperative ROM at 6 months after TKA compared to the medial parapatellar approach [38]. We evaluated the knee ROM 12 months after the TKA, and the two groups were not significantly different.

Findings of an RCT comparing the functional outcomes of the subvastus and medial parapatellar approaches in 76 patients showed that the Oxford Knee Scores (OKS) were not significantly different between the two groups [20]. However, the knee functional scores at the 12 and 18‐month follow‐ups were better in the medial parapatellar group [20]. A recent study by Aladraii evaluated the functional outcome of TKAs with subvastus and medial parapatellar approaches and found that patients in the subvastus group had better WOMAC and OKS scores at 3 and 6‐month follow‐ups. However, the two groups showed no significant difference at the 12‐month follow‐up [39]. We found that both groups showed improvements in pain, physical function, overall score of WOMAC, and Knee functional score one year after the TKA; however, the reductions were more significant in the subvastus group.

Our study evaluated the long‐term outcomes of the medial parapatellar and subvastus approaches. The findings of our study demonstrated that after 10–15 years following TKA, the VAS pain score and functional scores of WOMAC and KSS were not significantly different between the two approaches. In the medial parapatellar approach, the vastus medialis is separated from the patella in the medial parapatellar approach by intratendinous incision, which results in disruption of blood supply to the patella, which increases the risk of patellar fractures [8, 40]. The subvastus arthrotomy offers early strength advantages and potential anatomical and vascular benefits, making it a viable alternative to the traditional parapatellar approach for TKA. However, the subvastus approach is not recommended in patients with limited knee ROM, short stature, obesity, large thigh girth, and patella baja due to the potential limitation of the surgical exposure that affects the outcome of the surgery [41, 42, 43]. While the subvastus approach for TKA offers significant benefits in terms of early recovery and rehabilitation, its application is not universal. It is essential to note that the standard medial parapatellar approach is equally effective in achieving positive outcomes in TKA in the long‐term follow‐up.

As it is a retrospective study, the main limitation of our study was that the division of patients between the two groups in this study was not randomised. We suggest multicentric randomised studies with larger sample sizes, assessment of other clinical characteristics, and comparison with other minimally invasive approaches for definite clinical recommendations.

## CONCLUSION

In conclusion, our findings indicated that TKA using the subvastus approach resulted in better functional scores one year postoperatively. The subvastus approach was associated with improved pain and a shorter time to achieve active SLR at the early postoperative stage, although long‐term outcomes did not significantly differ between the two approaches.

## AUTHOR CONTRIBUTIONS

All authors contributed to the study's conception and design. Pooya Hosseini‐Monfared, Alireza Mirahmadi, Shayan Amiri, and Mohammad Hossein Ghafouri participated in data gathering. Pooya Hosseini‐Monfared and Alireza Mirahmadi performed data analysis. Pooya Hosseini‐Monfared, Alireza Mirahmadi, Shayan Amiri, and Mohammad Hossein Ghafouri drafted the manuscript. Reza Minaie and Seyed Morteza Kazemi revised the manuscript. All authors read and approved the final version of the manuscript.

## CONFLICT OF INTEREST STATEMENT

The authors declare no conflict of interest.

## ETHICS STATEMENT

The study received ethical approval from the Institutional Review Board (IR.SBMU.MSP.REC.1397.694).

## Data Availability

The data used and analysed during the current study are available from the corresponding author upon reasonable request.
